# Impact of Surgeon’s Experience in Rigid versus Elastic MRI/TRUS-Fusion Biopsy to Detect Significant Prostate Cancer Using Targeted and Systematic Cores

**DOI:** 10.3390/cancers14040886

**Published:** 2022-02-10

**Authors:** Magdalena Görtz, Joanne Nyaboe Nyarangi-Dix, Lars Pursche, Viktoria Schütz, Philipp Reimold, Constantin Schwab, Albrecht Stenzinger, Holger Sültmann, Stefan Duensing, Heinz-Peter Schlemmer, David Bonekamp, Markus Hohenfellner, Jan Philipp Radtke

**Affiliations:** 1 Department of Urology, University Hospital Heidelberg, 69120 Heidelberg, Germany; joannenyaboe.nyarangi-dix@med.uni-heidelberg.de (J.N.N.-D.); lars.pursche@med.uni-heidelberg.de (L.P.); viktoria.schuetz@med.uni-heidelberg.de (V.S.); philipp.reimold@med.uni-heidelberg.de (P.R.); markus.hohenfellner@med.uni-heidelberg.de (M.H.); radtke.janphilipp@googlemail.com (J.P.R.); 2 Helmholtz Young Investigator Group ‘Multiparametric Methods for Early Detection of Prostate Carcinoma’, German Cancer Research Center (DKFZ), 69120 Heidelberg, Germany; 3 Institute of Pathology, University Heidelberg, 69120 Heidelberg, Germany; constantin.schwab@med.uni-heidelberg.de (C.S.); albrecht.stenzinger@med.uni-heidelberg.de (A.S.); 4 Division of Cancer Genome Research, German Cancer Research Center (DKFZ) and German Cancer Consortium (DKTK), 69120 Heidelberg, Germany; h.sueltmann@dkfz-heidelberg.de; 5 Section of Molecular Urooncology, Department of Urology, University of Heidelberg School of Medicine, 69120 Heidelberg, Germany; stefan.duensing@med.uni-heidelberg.de; 6 Department of Radiology, German Cancer Research Center (DKFZ), 69120 Heidelberg, Germany; h.schlemmer@dkfz-heidelberg.de (H.-P.S.); d.bonekamp@dkfz-heidelberg.de (D.B.); 7 Department of Urology, University Hospital Düsseldorf, 40225 Düsseldorf, Germany

**Keywords:** elastic biopsy, fusion-targeted prostate biopsy, learning curve, magnetic resonance imaging, prostate cancer, rigid biopsy

## Abstract

**Simple Summary:**

For the transfer of suspicious lesions in magnetic resonance imaging (MRI) to ultrasound in prostate fusion biopsy, biopsy platforms can be distinguished by rigid or elastic image registration. This study evaluates the detection rate of these different platforms for transperineal fusion-guided prostate biopsy to detect clinically significant prostate cancer under consideration of the surgeon’s learning curve. In our cohort, rigid and elastic registration systems showed a similar prostate cancer detection rate in experienced surgeons, whereas novices seem to benefit from rigid fusion. In the total cohort, targeted fusion biopsy with a rigid registration system outperformed elastic registration target biopsy with a superior significant prostate cancer detection rate, each compared to systematic saturation biopsy. Thus, rigid target biopsy aided in reducing targeting errors that result in missing MRI-visualized significant prostate cancer. These results can provide valuable decision support in selecting a biopsy fusion platform to increase the detection rate and risk stratification of prostate cancer, especially at the beginning of the surgeon’s learning curve.

**Abstract:**

Multiparametric magnetic resonance imaging (mpMRI) and MRI/ultrasound fusion-targeted prostate biopsy (FB) have excellent sensitivity in detecting significant prostate cancer (sPC). FB platforms can be distinguished by rigid (RTB) or elastic image registration (ETB). We compared RTB and ETB by analyzing sPC detection rates of both RTB and ETB at different stages of the surgeons’ learning curve. Patients undergoing RTB between 2015–2017 (*n* = 502) were compared to patients undergoing ETB from 2017–2019 (*n* = 437). SPC detection rates were compared by Chi-square-test on patient-basis. Combination of transperineal systematic biopsy and each TB served as reference and sub-analyses were performed for different grades of surgeon’s experience. In the RTB subgroup, 233 men (46%) had sPC, compared to 201 (46%) in the ETB subgroup. RTB alone detected 94% of men with sPC and ETB 87% (*p* = 0.02). However, for at least intermediate-experienced surgeons (>100 FB), no differences occurred between RTB and ETB. In the total cohort, at least intermediate-experienced surgeons detected significantly more sPC (10%, *p* = 0.008) than novices. Thus, targeted transperineal MRI/TRUS-FB with a RTB registration system showed a similar sPC detection rate to ETB in experienced surgeons but a superior sPC detection rate to ETB in the total cohort. Low-experienced surgeons seem to benefit from RTB.

## 1. Introduction

Multiparametric magnetic resonance imaging (mpMRI) and mpMRI-guided biopsies of the prostate have an important role in prostate cancer (PC) detection as they improve the detection of clinically significant PC (sPC) while mitigating insignificant PC [1,2,3,4]. The risk assessment by MRI prior to biopsy followed by MRI-guided biopsy has been found to be superior to the standard approach using 12-core transrectal ultrasound (TRUS)-guided biopsy [2]. Demonstrating superior detection rates of sPC, mpMRI has been recommended to be used regularly prior to prostate biopsy even in biopsy-naïve patients [1,2,4]. Standardized MRI interpretation using the Prostate Imaging Reporting and Data System (PI-RADS) classification is used to assess mpMRI on a scale of one to five, with PI-RADS scores ≥3 undergoing targeted cores [5,6].

Performing targeted biopsy (TB) under direct MRI guidance is expensive, time-consuming, and not widely available. On the other hand, TRUS-guided prostate biopsy, despite the lower detection rates, has the advantages of speed, lower costs, and wider availability [7]. Thus, cognitive or software-guided MRI/TRUS fusion biopsy has become the standard diagnostic procedure to perform MRI-guided biopsy [8,9]. Recent literature like the FUTURE trial demonstrated that there is not a significant disadvantage between the three MRI-based biopsy techniques [10]. Due to a negligible rate of sepsis, the transperineal approach is recommended over transrectal prostate biopsy. However, prostate biopsy can lead to complications such as bleeding and urinary retention [11].

While MRI/TRUS-TB is more accurate at detecting sPC than 12-core TRUS-biopsy, there is a significant learning curve in MRI/TRUS fusion biopsy. According to Kasabwala et al., a significant improvement in TB accuracy occurs up to 98 cases of prostate TB [12]. Precise diagnosis is the prerequisite to not miss patients with sPC and to achieve stratification of patients into risk groups, leading to optimal treatment decisions according to the disease stage. To accurately assess patients’ risk of tumor progression, the result of prostate biopsy is commonly used to stratify patients into low- and high-risk groups [13].

Computerized MRI/TRUS image registration, which consists of an overlay of MRI-detected lesions and corresponding TRUS images, allows clinicians to perform TB using co-registered images and live ultrasound [7]. It combines the benefits of mpMRI with the identified suspicious lesions with the versatility of ultrasound in an outpatient setting [14]. Registration is used to bring the separate images from MRI and ultrasound into spatial alignment for the prostate biopsy using fusion software. There are several commercially available software-assisted MRI/TRUS fusion platforms using different methods of registration (rigid or elastic) to fuse MRI and ultrasound. Rigid registration enables the surgeon to manually rotate the MRI and TRUS images with respect to each other to produce the best alignment between the images, although the images themselves do not change [15]. In contrast, elastic registration uses a software algorithm to compensate for changes in the segmented prostate shape, which can occur between the preoperative MRI and intraoperative imaging during prostate biopsy [16]. The surgeon can still rotate the rigid images initially to create alignment, but after elastic transformation is applied the semiautomated software algorithm deforms the segmented margin of the MRI to fit the segmented real-time ultrasound margin [15].

Previous studies showed the feasibility of MRI/TRUS image registration-based TB, but the value of rigid TB (RTB) versus elastic TB (ETB) during fusion-guided prostate biopsy has been poorly studied under consideration of the surgeon’s learning curve. It is unclear which fusion approach is providing most benefit to surgeons at the beginning of the learning curve to achieve optimal sPC detection.

This study aims to evaluate the role of surgeon’s experience in combination with the different TB approaches to detect sPC. We analyzed the surgeon’s experience with respect to two commercially available systems using either transperineal RTB or ETB for sPC detection, each compared to systematic saturation biopsy (SB).

## 2. Materials and Methods

### 2.1. Study Population

**Figure 1:**
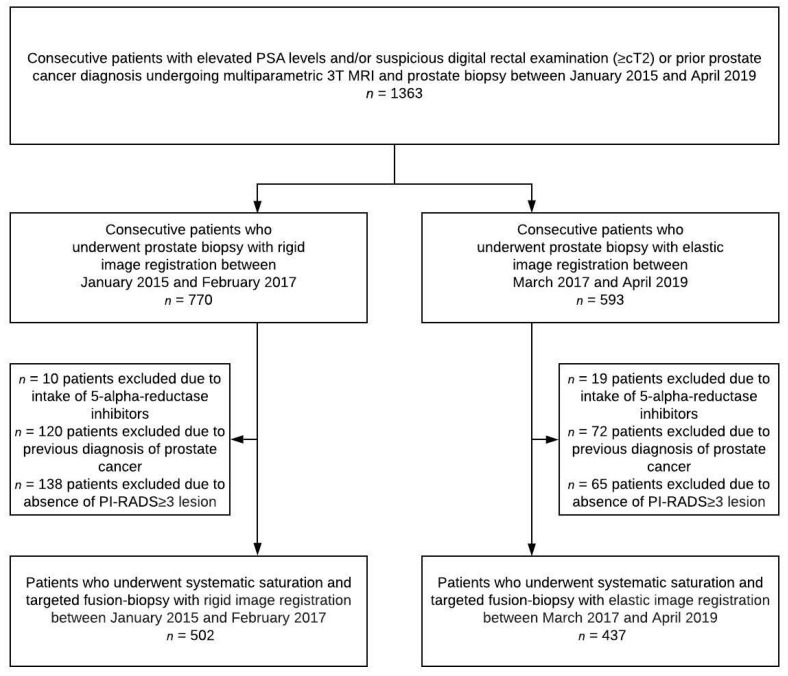
Flow chart for subgroups. MRI = magnetic resonance imaging; PI-RADS = Prostate Imaging-Reporting and Data System; PSA = prostate specific antigen.

A total of 1363 consecutive men received MRI-TRUS fusion-biopsy at the University Hospital Heidelberg between January 2015 and April 2019 due to suspicion of PC with a prostate specific antigen (PSA) level ≥4 ng/mL and/or suspicious digital rectal exam (DRE) (Figure 1). MpMRI was carried out on a single 3 Tesla MRI system (Magnetom Prisma, Siemens Healthcare, Erlangen, Germany) at the German Cancer Research Center. After excluding men who took 5-alpha-reductase inhibitors, had a previous diagnosis of PC and/or a negative mpMRI (PI-RADS < 3), 939 men were included in this study. This study includes previously published patient data [17,18]. Data were collected prospectively and institutional review board approval was provided (S-156/2018).

### 2.2. MRI Analysis

All patients underwent prebiopsy mpMRI without an endorectal coil. All MRI scans were acquired according to the PI-RADS recommendations using a standard multichannel body coil and integrated spine phased array coil [5,19]. Axial, coronal, and sagittal T2-weighted images, echo-planar imaging diffusion-weighted images (DWI) including apparent diffusion coefficient (ADC) maps, and dynamic-contrast enhanced images were acquired. DWI was performed using *b*-values 50, 500, 1000, and 1500 s/mm^2^. Clinical mpMRI reporting was performed during clinical routine by board-certified radiologists according to PI-RADS. Radiologists with at least five years of experience in prostate MR image interpretation interpreted the studies. All examinations were reviewed again in an interdisciplinary conference prior to biopsy for quality assurance and all radiologists participated in regular retrospective review of MRI reports and biopsy results.

### 2.3. Biopsy

Men underwent transperineal grid-directed biopsy performed under general anesthesia with rigid software registration using the MedCom BiopSee platform (MedCom, Darmstadt, Germany) from January 2015 until February 2017. In March 2017, the software-assisted MRI/TRUS fusion platform was changed at our institution. From March 2017 until April 2019, transperineal grid-directed biopsy was performed with elastic software registration using the UroNav platform (Philips Invivo, Gainsville, FL, USA). In all cases, TB of MRI-suspicious lesions was performed first, followed by SB according to the Ginsburg protocol and scheme, as previously described [20,21]. Only trained and qualified urologists with a significant experience of at least 50 MRI-TRUS fusion biopsies performed the prostate biopsies in our cohort. In addition, experience of the respective 17 biopsy surgeons was stratified according to Kasabwala et al. [12] in 50–100, 100–200, and >200 TB (Table 1).

### 2.4. Pathology

Targeted and systematic cores were fixed, paraffin-embedded, and analyzed separately under the supervision of a dedicated uropathologist (AS) according to ISUP (International Society of Urological Pathology) standards [22,23]. sPC was defined as Gleason score (GS) ≥3+4.

### 2.5. Statistical Analysis

Patient, MRI, and biopsy data were analyzed descriptively according to START [24].

The detection difference of RTB and ETB was estimated with the use of a Chi-square-test for independent samples. We performed statistical analysis on patient-basis. To further evaluate the magnitude of differences in detection rates among the different biopsy approaches for sPC, we calculated rate differences using 95% confidence intervals (CI) according to Altman for independent and Tango for dependent variables. All tests were performed two-sided with a significance level of 5%. Bonferroni-Holm correction was used for multiple testing to reduce the probability of alpha error accumulation. Statistical analyses were performed using R version 3.5.0 (R Foundation for Statistical Computing, Vienna, Austria), SPSS version 22 (IBM, Armonk, NY, USA), and MedCalc version 14 (MedCalc Software, Ostend, Belgium).

## 3. Results

In the study cohort of 939 men, sPC was detected in 434 of 939 (46% of all men) patients through the combination of TB and extended SB with more than 20 systematic biopsies of the prostate [20]. In total, TB alone would not have detected 39 of 434 (9%) sPC, while SB alone would have missed 47 of 434 (11%) sPC.

We identified a group of 502 patients undergoing RTB from January 2015 until February 2017 and a group of 437 patients undergoing ETB from March 2017 until April 2019. Patients’ clinical characteristics, MRI findings, and biopsy results for RTB vs. ETB are given in Table 1. In the RTB group, 839 PI-RADS ≥ 3 lesions occurred and underwent subsequent RTB, whereas 766 PI-RADS ≥ 3 lesions were targeted biopsied using ETB. The two groups were similar in the detection rates of sPC, age, and PSA level. Within the RTB group, significantly more patients had suspicious DRE findings (*p* = 0.02) and showed a smaller median prostate volume, as compared to the ETB group (*p* = 0.001). The number of SB and TB cores per target lesion were not significantly different.

**Table 1:** Patient characteristics, magnetic resonance imaging findings, and biopsy cores for the rigid and elastic registration patient groups according to START protocol [24]. DRE = digital rectal examination; IQR = inter-quartile range; PI-RADS = Prostate Imaging-Reporting and Data System; PSA = prostate specific antigen.

	Rigid System	Elastic System	*p* Value
Men included in study, *n*	502	437	
Men with significant prostate cancer, *n* (%)	233 (46)	201 (46)	0.39
Men with insignificant prostate cancer, *n* (%)	81 (16)	59 (14)	0.39
Age, years, median (IQR)	65 (58–71)	65 (59–70)	0.30
PSA level, ng/mL, median (IQR)	7.7 (5.4–11.6)	7.6 (5.4–10.8)	0.38
Suspicious DRE finding (≥T2), *n* (%)	176 (35)	121 (28)	0.02
Prostate volume, mL, median (IQR)	44 (31–62)	50 (34–73)	0.001
PSA density, ng/mL^2^, median (IQR)	0.17 (0.11–0.27)	0.15 (0.09–0.25)	0.02
Biopsies per patient, median (IQR)	29 (26–33)	34 (29–39)	1.00
Systematic biopsies per patient, median (IQR)	24 (20–26)	25 (22–29)	0.89
Targeted biopsies per lesion, median (IQR)	4 (3–5)	5 (4–6)	0.84
Number of lesions PI-RADS ≥ 3, *n*	839	766	
Patients with one PI-RADS ≥ 3 lesion, *n* (%)	272 (54)	205 (47)	
Patients with two PI-RADS ≥ 3 lesions, *n* (%)	157 (31)	158 (36)	
Patients with three PI-RADS ≥ 3 lesions, *n* (%)	48 (10)	57 (13)	
Patients with ≥ four PI-RADS ≥ 3 lesions, *n* (%)	25 (5)	17 (4)	
Overall PI-RADS score 3 lesions, *n* (%)	367 (44)	310 (40)	
Overall PI-RADS score 4 lesions, *n* (%)	333 (40)	332 (43)	
Overall PI-RADS score 5 lesions, *n* (%)	139 (17)	124 (16)	
Number of investigators performing biopsy, *n*	17	17	
Investigator experience > 200 biopsies, *n* (%)	4 (24)	5 (29)	
Investigator experience 100–200 biopsies, *n* (%)	7 (41)	5 (29)	
Investigator experience < 100 biopsies, *n* (%)	6 (35)	7 (41)	
Number of biopsies performed by investigators with >200 biopsies, *n* (%)	146 (29)	162 (37)	0.009
Number of biopsies performed by investigators with 100–200 biopsies, *n* (%)	130 (26)	201 (46)	<0.001
Number of biopsies performed by investigators with <100 biopsies, *n* (%)	226 (45)	74 (17)	<0.001

RTB alone identified 220 of the 233 (94%) men with sPC that were detected by the combination of SB and TB. ETB alone identified 175 of the 201 (87%) men suffering from sPC. Consequently, 13 (6%) men were diagnosed by the SB part alone in the RTB and 26 (13%) men in the ETB subgroup.

When surgeon’s experience was analyzed, surgeons with lower experience (50–100 biopsies) had a lower sPC detection rate with ETB as compared to RTB (82% versus 94%). However, this difference reached no statistical significance (*p* = 0.05). Results of intermediate (100–200 biopsies) and highly experienced surgeons (>200 biopsies) are comparable for both approaches (Table 2).

Results of the influence of biopsy experience in the overall cohort are given in Table 3. Here, at least intermediate-biopsy experience demonstrated a significant higher chance of 10% to detect sPC (95% versus 85%, *p* = 0.008) as compared to low-experienced surgeons. However, sPC detection rate of high-versus intermediate-experienced surgeons did not show a significant difference (*p* = 0.58).

If sPC is detected in SB but not in TB, this can be due to sPC missed in TB due to fusion errors or because a tumor lesion located elsewhere in the prostate was not detected on MRI. All men with sPC detection in SB but not in TB were examined to determine if the positive cores of SB were in the position of the MRI lesion. The lesions missed by TB in these 39 men had indeed positive SB cores in close proximity to the MRI lesions. Consequently, sPC was missed by TB due to FB registration error and not due to out-of-field positive biopsies. When RTB and ETB were compared in the total cohort, the ability of RTB to detect sPC was significantly higher as compared to ETB (*p* = 0.02, Table 2).

**Table 2:** Detection of significant prostate cancer by targeted fusion-biopsy and rigid vs. elastic registration and subanalyses of different surgical experience groups. sPC = significant prostate cancer.

	Rigid Targeted Biopsy (*n* = 233 Men with sPC)	Elastic Targeted Biopsy (*n* = 201 Men with sPC)	Difference (Confidence Interval)	*p* Value
sPC detected by TB, %	220/233 (94)	175/201 (87)	7 (1.5–12)	0.02
Highly experienced surgeons	79/86 (92)	55/59 (92)	0 (−9.0–11)	1.00
Intermediate experienced surgeons	105/109 (96)	47/51 (92)	4 (−3.8–15)	0.58
Low experienced surgeons	36/38 (95)	74/91 (82)	13 (−11–24)	0.05

**Table 3:** Detection of significant prostate cancer by targeted fusion-biopsy and stratified to different experience combined for both fusion-biopsy approaches. sPC = significant prostate cancer.

	Highly Experienced (*n* = 145)	Intermediate Experienced (*n* = 160)	Difference (Confidence Interval)	*p* Value
sPC detection (%)	134/145 (92)	152/160 (95)	−3.0 (−9.1–2.7)	0.58
	**Intermediate Experienced (*n* = 160)**	**Low Experienced** **(*n* = 129)**	**Difference** **D(Confidence Interval)**	***p* Value**
sPC detection (%)	152/160 (95)	110/129 (85)	10 (3.1–18)	0.008

For patients with missed sPC in TB due to registration inaccuracy, the number of PI-RADS lesions and their locations are given in Table 4.

**Table 4:** Characteristics of patients with significant prostate cancer missed in targeted fusion biopsy. PI-RADS = Prostate Imaging-Reporting and Data System.

	Rigid System	Elastic System	*p* Value
Number of patients	13	26	
Prostate volume, ml, median (IQR)	42 (31–62)	45 (28–70)	0.43
Number of lesions PI-RADS ≥ 3	27	43	
PI-RADS score 3 lesion, *n* (%)	11 (41)	9 (21)	
PI-RADS score 4 lesion, *n* (%)	11 (41)	27 (63)	
PI-RADS score 5 lesion, *n* (%)	5 (19)	7 (16)	
Location of the lesion in the peripheral zone, *n* (%)	17 (63)	37 (86)	
Location of the lesion in the transitional zone, *n* (%)	9 (33)	6 (14)	
Location of the lesion in the anterior stroma, *n* (%)	1 (4)	0 (0)	
Volume of the lesion, mL, median (IQR)	0.3 (0.1–0.8)	0.4 (0.2–0.8)	0.95

## 4. Discussion

The value of TB is widely acknowledged as MRI/TRUS fusion-biopsies detect more high-risk prostate tumors than conventional 12-core TRUS biopsies [25]. Since mpMRI has been recommended to be used prior to prostate biopsy [2], the challenge for clinicians is to identify the most accurate technique to perform MRI-guided biopsies.

Several studies with different fusion systems showed that some sPC are missed in TB and that the combination of systematic TRUS biopsy and TB detect more sPC than TB alone [4,21]. This finding was confirmed in our study in both the RTB and the ETB group. Since small targeting errors in the range of 1 to 2 mm can result in missing a MRI-visualized sPC, it is important to achieve the best possible registration and fusion of images [26]. To overcome this issue, two options are possible. First, some authors propagate the utilization of ‘target saturation’, the application of in median eight to ten cores to the lesion and the penumbra [27,28]. This is also supported by the PI-RADS v2.1 steering committee [5]. The second option is to precise the TB technique. There are several providers with different fusion platforms, which can be distinguished according to ETB vs. RTB and transperineal vs. transrectal access. In our institution, transperineal biopsy is performed with a median of four targeted cores per lesion. A minimum of three to four targeted biopsy cores are recommended to be obtained from PI-RADS 3 and 4 lesions to ensure accurate diagnosis [29].

In this study, we compared two common commercially available systems using different approaches to perform fusion of MRI and TRUS images (MedCom BiopSee for RTB and Invivo UroNav for ETB), focusing in particular on different stages of the surgeon’s learning curve. To our knowledge this study is the first to compare the two techniques of computer-assisted MRI/TRUS ETB vs. RTB for transperineal prostate biopsies in a large patient cohort and under consideration of the learning curve. Our analysis revealed two major results.

First, the level of experience significantly influenced the sPC detection rate. Second, in our cohort, RTB had an improved sPC detection rate compared to ETB. The difference seen in the overall cohort between sPC detection in RTB and ETB (*p* = 0.02) is attenuated in highly experienced surgeons (*p* = 1.00). In particular, surgeons with low experience had lower detection rates as compared to intermediate and highly experienced surgeons in ETB. Thus, surgeons with low experience seem to profit from RTB to achieve an optimal sPC detection rate. This is an interesting finding, as it is a common opinion that ETB platforms mitigate gland and lesion deformation [15], therefore offering benefits especially in surgeons within or at the beginning of the learning curve. However, a recent analysis by Sokolakis et al. demonstrated that rigid registration platforms might be easier to use compared to elastic registration platforms among urologists at the beginning of the learning curve [30]. It can only be speculated whether the sole reliance of the inexperienced surgeon on the software algorithm leads to larger fusion errors. However, both FB methods have their advantages: rigid registration does not accommodate for the deformation of the prostate gland, but maintains the true patient anatomy detected on MRI, while elastic registration maximizes fusion image alignment.

We could demonstrate that in the overall cohort, intermediate surgeon’s experience with at least 100 TB performed leads to a significantly higher sPC detection rate. This is comparable to Kasabwala et al. and Halstuch et al., demonstrating a significant improvement of the sPC detection rate after 98 and 119 TB, respectively [12,31]. In addition, our result showing a 10% increase of sPC detection for experienced surgeons, reproduced the results of Calio et al., demonstrating a 13% increase in sPC detection over time [32]. We could also demonstrate that in both ETB and RTB approaches, the learning curve flattens after reaching intermediate experience, which is higher compared to Gaziev et al. with 70 cases and Mager et al. with 63 cases [33,34].

Analyzing the results of our monocentric cohort, we could demonstrate that in a setting with experienced biopsy surgeons, there is no statistically significant difference in the sPC detection rate of RTB versus ETB (92% sPC detection rate each), although RTB alone detected 94% as compared to 87% for ETB in the total cohort. Our results are in contrast to previous studies [7,35,36]. In detail, Venderink et al. performed a meta-analysis comparing elastic versus rigid fusion platforms [36]. They could first demonstrate that both TB approaches had a 40–45% improvement in sPC detection as compared to SB, but second that there was no statistically significant difference between ETB and RTB. This is in contrast to our study, demonstrating a 7% higher detection rate for RTB. However, Venderink et al. did not perform a stratified subgroup analysis with respect to the learning curve.

In addition, in our cohort the benefit for RTB versus extended SB was 3% and therefore demonstrated a 15% improvement over years as compared to RTB alone in 2015 [21]. We could not demonstrate a difference in the comparison of ETB versus extended SB (both 87%). While this is slightly lower as compared to Arsov et al. using the Uronav platform demonstrating a 5% increase in sPC detection, we acknowledge that this is also improved over time as compared to TB alone [21,37]. However, we also acknowledge that the 5% increase demonstrated by Arsov et al. was compared to 12-core TRUS biopsy, which demonstrated lower sPC detection rates as compared to extended SB [38].

Another clinical study compared the transrectal rigid and elastic registration method (using Esaote^®^ and Koelis^®^) by determining the PC detection rates of TB and random biopsy, demonstrating no significant difference in the detection rate between RTB and ETB. However, the study cohort size was limited with <100 patients having PC in each group [7].

The overall detection rate must be further discussed as the detection rate was relatively high for all biopsy approaches. Both the RTB and ETB rates were comparable to a recent analysis by Ahdoot et al., demonstrating a 92% detection rate for ETB using the Uronav platform [39]. As compared to a recent analysis of the extended SB scheme of the Ginsburg study, an improved detection rate of SB with 87–91% compared to 81% has been demonstrated in this study [20,28]. This higher detection rate limits the improvement by both TB approaches as compared to extended SB.

One strength of this study is that it is, to our knowledge, the first large analysis of men that have undergone transperineal RTB or ETB under consideration of the surgeon’s learning curve. We acknowledge that this was an allocation over time and not prospectively randomized. However, our study represents a real-life scenario and the overall surgeon’s performance in this single-center analysis of an experienced center is comparable to other specialized centers.

Our study has some limitations to discuss. It includes all potential biases that are inherent with a non-randomized study design, treating patients in different periods of time. Studies with prospectively randomized patient cohorts should be performed to confirm our results.

A further limitation is that only two of many fusion biopsy platforms were used. The generalizability of our results may be restricted since elastic registration algorithms differ slightly among platforms. However, the results are comparable to previous studies using the same fusion platforms [21,36,37,39,40].

In addition, in our study the reference standard was SB and TB rather than radical prostatectomy (RP). However, a RP cohort would introduce bias by excluding many patients from the analysis. Furthermore, the approach of transperineal MRI TB in combination with SB has been validated previously by correlation with RP specimen, demonstrating that 97% of sPC have been detected by combined TB and SB [21].

In our institution, SB with an extended number of biopsy cores is performed for maximum diagnostic safety. The addition of saturated SB can achieve high PC detection rates and can compensate for fusion errors, regardless of the TB approach. However, the optimal biopsy strategy would be highly sensitive for sPC with only a limited number of biopsy cores to avoid potential side effects of the biopsy [41] and potential overdetection of clinically insignificant PC. If a prostate biopsy with a low number of biopsy cores is performed, a high diagnostic accuracy must be ensured. Furthermore, a registration error that is as small as possible is essential for future developments like radiogenomic analyses of MRI lesions.

## 5. Conclusions

In our cohort, targeted transperineal MRI/TRUS-TB with a rigid image registration system showed a significantly higher sPC detection rate than elastic TB. Our results demonstrate no difference between RTB and ETB for surgeons with experience >100 biopsies, whereas less experienced surgeons seem to benefit from RTB. Omitting SB would come at a risk of 6–13% missing sPC, depending on the TB approach. Depending on the surgeon’s experience, 5–7% of sPC are missed by TB in intermediate and highly experienced surgeons, whereas low experience is associated with missing sPC in 15% of men, suggesting a learning curve of TB up to approximately 100 cases.

## Data Availability

The data presented in this study are available on request from the corresponding author.

## Abbreviations

ADC	apparent diffusion coefficient
CI	confidence interval
DRE	digital rectal examination
DWI	diffusion-weighted images
ETB	elastic targeted fusion-biopsy
FB	fusion biopsy
GS	Gleason score
ISUP	International Society of Urological Pathology
mpMRI	multiparametric magnetic resonance imaging
PC	prostate cancer
PI-RADS	Prostate Imaging Reporting and Data System
PSA	prostate specific antigen
RP	radical prostatectomy
RTB	rigid targeted fusion-biopsy
SB	systematic saturation biopsy
sPC	significant prostate cancer
TB	targeted fusion-biopsy
TRUS	transrectal ultrasound
